# Being There: Exploring Virtual Symphonic Experience as a Salutogenic Design Intervention for Older Adults

**DOI:** 10.3389/fpsyg.2021.541656

**Published:** 2021-12-02

**Authors:** Meara H. Faw, Taylor Buley, Laura Healey Malinin

**Affiliations:** ^1^Department of Communication Studies, Colorado State University, Fort Collins, CO, United States; ^2^Department of Design and Merchandising, College of Health and Human Sciences, Colorado State University, Fort Collins, CO, United States

**Keywords:** dementia, Alzheimer’s disease, healthy aging, virtual reality, psychosocial intervention, salutogenic design

## Abstract

This co-design study examined salutogenic potential of mobile virtual reality (VR) experiences as an alternative to participation in a community-based symphonic engagement program (B Sharp), previously found to benefit people with dementia (PWD) and their informal caregivers. Six focus groups were conducted with sixteen adults aged 76–90; three participants had dementia, and two were informal spousal caregivers. No participants had prior VR experience. The study assessed the feasibility of replicating the community-based-arts program in VR, with the goal of enhancing its salutogenic qualities (e.g., positive distraction, engagement, and social connection). Video-recordings of participants while using a mobile head-mounted display (HMD) were analyzed using qualitative thematic analysis to compare perceptions of different virtual experiences, including replication or enhancement of B Sharp and a campus tour. Findings suggest participants had positive perceptions of enhanced VR experiences with no adverse effects, although PWD were less enthusiastic and HMD usability was complicated by eyewear use and comfort with technology. Participants reacted most favorably to the enhanced symphonic experience, where they were “virtually” onstage during the performance, suggesting unique experiences beyond what is possible in the real world have the greatest potential for deep immersion for older adults. Results suggest VR has strong potential to replicate and enhance salutogenic qualities of community-based programming by enabling greater access to experiences for older adults and by increasing enjoyment and engagement through experiences not otherwise feasible. Furthermore, this study illuminates advantages of a user-centered, co-design approach when developing VR experiences with community partners and older adults.

## Introduction

Age-related cognitive decline is a significant global health challenge (World Health Organization, 2015). Cognitive function, essential for independent living and intertwined with physical health (Li and Lindenberger, 2002; Gross et al., 2011), typically declines from the 7th decade^1^ (Li et al., 2004). However, with dementia, it deteriorates rapidly and at an earlier age (Bayles et al., 1987). Dementia is a degenerative condition involving cognitive impairments that interfere with a person’s ability to live independently and reduce their quality of life (McKhann et al., 2011). Pharmaceuticals intended to slow dementia-related decline have limited efficacy, problematic side effects, and poor compliance (Serafino, 2018; Davalos et al., 2019). Conversely, participation in enriching activities (e.g., music and arts programming) have shown delayed or reduced functional decline without adverse effects (Raglio et al., 2012; Dyer et al., 2018; Davalos et al., 2019).

Our team studies impacts of community-based arts programming (CBAP), including symphony (Davalos et al., 2019; Faw et al., 2021), dance, theater, and craft (Griggs et al., 2020) experiences on cognitive function and quality of life for people with dementia (PWD) and their caregiving partners (CPs). Our symphonic program, B Sharp, involves PWDs and CPs who receive symphony season tickets. Dyads complete cognitive assessments and quality of life surveys at the beginning and end of each season. Results from the program’s first year found overall cognitive performance improved for PWD over the 10-month season (Davalos et al., 2019), with greater improvements for more frequent attendees. Additionally, dyads expressed high motivation to participate due to restorative and engaging program aspects, including a sense of “being away” and social connection (Faw et al., 2021). Despite program benefits, some dyads reported challenges related to transportation, performance timing, and other barriers to attendance. These findings, along with challenges of the recent COVID-19 pandemic, underscore the need for programming that can be delivered while adhering to safe social distancing and in accessible environments (Armitage and Nellums, 2020).

VR is an increasingly-accessible and moderately-priced technology that can enable frequent exposure to symphonic performances and provide access to those unable to attend live events. VR head-mounted displays (HMDs) enable dynamic immersion in digitally created experiences (Parsons, 2015) and are rapidly gaining popularity in clinical and research settings (Parsons, 2015; Hoffman et al., 2019; Kourtesis et al., 2019). Therapeutic applications include the use of VR to treat phobias (Diemer et al., 2013; Malbos et al., 2013), post-traumatic stress disorder (Rothbaum et al., 2014; Norrholm et al., 2016; Beidel et al., 2019), psychotic disorders (du Sert et al., 2018; Pot-Kolder et al., 2018), stroke (Threapleton et al., 2017), and pain management (Gomez et al., 2017; Kourtesis et al., 2019). VR applications have also been used to enhance cognition for PWD (Optale et al., 2010; Man et al., 2012; Manera et al., 2016; Doniger et al., 2018; Gamito et al., 2018).

Although these studies suggest VR’s therapeutic benefits, its salutogenic potential remains underexplored. Salutogenesis emphasizes examining factors that support health as opposed to focusing on causes of a disease (Antonovsky, 1979). Salutogenic design is an evidence-based approach to constructing environments that alleviate stress and promote increased engagement and social connection (Mazzi, 2020), thereby enhancing wellbeing, including specific strategies for older adults (Burzynska and Malinin, 2017) and PWD (Mobley et al., 2017). Preliminary evidence from a study by Man et al. (2012) suggests VR can improve objective memory in older adults at risk of developing dementia; however, a key aspect of salutogenic design is to create environments that people enjoy and choose to engage by incorporating human-centered, co-design processes (Burzynska and Malinin, 2017; Mobley et al., 2017; Tsekleves and Cooper, 2017; Cinderby et al., 2018). Co-design involves end-users as collaborators during the design and evaluation of products or services and is particularly useful for surfacing technological challenges and preferences specific to older adults (Sumner et al., 2021). Thus, a salutogenic approach differs from other VR programs. Currently, limited research examines whether older adults find VR enjoyable—an important motivator for participation—and how they perceive VR simulations versus enhanced experiences, which allow them to experience situations unfeasible in real life (Lee et al., 2019). Our co-design study explores the potential of VR to simulate real-world experiences as well as to enhance them, building upon salutogenic effects found in real-world CBAP.

The purpose of our study was to engage older adults, including PWD and CPs, in simulated and enhanced VR-prototype experiences to assess their perceptions. It can be challenging and costly to create VR experiences; thus, we consider this study one step in an iterative, co-design process. A primary goal was to get participant feedback early in the design process to identify affordances and constraints of VR features toward improving participant engagement in the final design. Our project sought to answer the following questions:

(1) How do older adults, including PWD and CPs, perceive simulated and enhanced VR experiences?
(2) To what extent do participants perceive salutogenic design qualities (e.g., social connection and/or enjoyment) in the simulated versus enhanced VR experiences?
(3) Do perceptions and experiences of PWD differ from other participants?
(4) What desires do older adults have for future VR experiences?

## Materials and Methods

This study was conducted in two parts. All procedures received ethical approval from an institutional review board. The first three focus groups compared experiences with 360° video of a symphony performance intended to *simulate* the B Sharp experience and an *enhanced* campus tour. All participants (*n* = 16) in six focus groups (Table 1) consented to video recording and answered open-ended questions. Based on feedback from focus groups 1–3, an *enhanced* symphonic video replaced the simulated symphony video for focus groups 4–6. In total, three VR experiences (Table 2) were examined across the six focus groups. All VR experiences were recorded with 4 k resolution.

**TABLE 1:** Focus group participant information.

**Focus group (length)**	**Location**	**Participants**	**VR experiences**
Focus group 1 (59:26)	Private meeting room at an independent living center	Fred (PWD), Anne (CP), Robert (PWD), Peggy (CP), and Ken *Note: Anne and Fred had met the research team previously through another research study.*	Replication symphony campus tour
Focus group 2 (51:12)	Private meeting room at an independent living center	Sheila, Evelyn, and Gene	Replication symphony campus tour
Focus group 3 (41:43)	Private meeting room at an independent living center	Margaret, Leann, and June (PWD)	Replication symphony campus tour
Focus group 4 (46:58)	Participant Leann’s private residence	Leann and Betty	Enhanced symphony campus tour
Focus group 5 (38:58)	Private meeting room at a local senior center	Carl and Hal	Enhanced symphony campus tour
Focus group 6 (55:54)	Private meeting room at a local senior center	Darla and Nora	Enhanced symphony campus tour

**TABLE 2:** Virtual reality experience descriptions.

**Experience**	**Goal**	**Description**
Replication symphony experience	To examine whether a VR experience *replicating* that of a typical symphony concert would be immersive and enjoyable	This experience featured an audience-viewpoint intended to *replicate* the real-world viewpoint of a typical B Sharp participant. 360° footage was recorded in front of the soundboard, located in the middle of the auditorium. Participants were exposed to approximately 10 mins of a single symphonic movement. The movement was selected for its engaging nature and higher volume level
Enhanced symphony experience	To examine whether a VR experience *enhancing* a symphony concert (by placing participants center stage in close proximity to the musicians) would be immersive and enjoyable	This 360° video was recorded on stage directly in front of the conductor, intending to *enhance* the B Sharp experience by surrounding the viewer with the musicians. Participants viewed approximately 8 mins of a symphonic movement that involved string instruments and was upbeat and rhythmic
Enhanced campus tour experience	To examine whether a VR experience that was more active (i.e., moving around campus) and featured enhanced experiences (i.e., flying across campus and hovering above buildings) would be immersive and enjoyable. This experience also helped the researcher evaluate if participants would experience adverse effects from a more active VR experience (such as motion sickness). It also prompted participants to consider the types of enhancements they might enjoy in VR	The campus tour was an 8-min video featuring many activities across a local college campus. It included VR enhancements that people would not typically experience in real life, such as riding a golf cart through campus areas and flying above campus. It also featured more interactive activities, such as throwing confetti at a graduation celebration

Participants wore Oculus Go HMDs, selected because they are stand-alone, lightweight, with integrated speakers and high visual resolution screens, and moderately priced ($500). The HMD uses a single LCD at 2,560 × 1,440 (which amounts to 1,280 × 1,440 per eye) with a refresh rate of 60 Hz and field of view of about 101°, which gives a display fidelity of 12.67 pixels per degree. The HMD is a three-degrees of freedom headset, tracking rotations along the X, Y, and Z axis. It does not use an interpupillary distance (IPD) adjustment wheel but does have spacers to accommodate eyeglasses.

### Participants

Participants, recruited via convenience sampling using flyers and in-person announcements at local senior living and recreation centers, could not have a history of seizures or severe motion sickness. In total, six focus groups were conducted with 16 participants aged 76–90 years (*M* = 83.00, *SD* = 4.40); one person participated in both parts. A majority (*n* = 11) indicated some vision and/or hearing impairments; those with impairments participated with appropriate corrective measures (e.g., glasses, hearing aids). Ten participants were female and three were diagnosed with advanced dementia (no longer capable of independent living). Two PWDs attended with their CPs. None of the participants had prior experience using VR. Two knew the researchers prior to study participation through another research study. Researchers obtained written, informed consent from all participants. Participants were given pseudonyms and researchers anonymized identifiable information.

### Focus Group Procedures

Focus groups lasted approximately 50 mins (range = 38:58–59:26 mins) and followed a semi-structured protocol (Table 3). Focus groups were led by the first and second author (two women: a faculty member with doctorate degree and a graduate student) and attended by research assistants. All members of the research team had extensive experience interacting with older adults and leading qualitative projects. One team member had significant experience in co-design approaches. Participants talked with each other during the focus group, and this cross-talk was a valuable component of the focus groups (Greenbaum, 1998). Participants were asked about their initial impressions of VR and the HMD. Participants were then instructed to put on the HMD and relay their opinions regarding its comfort. Then, research assistants helped them enter the symphonic VR experience. All participants engaged in the virtual experience at the same time. Participants then removed the headset and responded to several questions (see Table 3). Next, participants entered the headset again and participated in the VR tour, after which they provided thoughts about the experience and their comfort level. Finally, participants evaluated their general impressions of VR and their desires for future VR experiences. In total, participants spent 15–20 mins in VR during the focus groups.

**TABLE 3:** Focus group protocol and sample questions.

**Focus group phase**	**Goal**	**Sample questions**
Pre-engagement	Inform participants about the goals and purpose of the research; obtain informed consent Assess participant familiarity and comfort with VR Introduce the headset and have participants try it on Gather initial impressions of the headset	How would you describe your comfort level with technology? When your hear the term “virtual reality”, what comes to mind? Have you ever tried VR before? What do you think about this headset? What do you notice about it? How did the headset feel?
Post-symphony VR experience questions	Assess participant responses to the VR experience Evaluate for positive/negative outcomes from engaging in the VR experience	How was your experience? What do you most remember about your VR experience? What did you like about it? What did you dislike about it? Did you feel immersed in the environment?
Post-campus tour VR experience questions	Assess participant responses to the VR experience Evaluate for positive/negative outcomes from engaging in the VR experience Compare participant perceptions of the symphony versus the tour experience	How was your experience? What do you most remember about your VR experience? What did you like about it? What did you dislike? Did you feel immersed in the environment?
De-brief questions	Evaluate participants’ overall impressions of VR Assess interest in future VR experiences	Did you enjoy this experience? Is this something you would want to do again? Would you feel comfortable using VR on your own? What about with training? Do you think others would enjoy this experience? If we were to design future VR experiences, what would you like for us to do?

### Data Analysis

Focus group video recordings and detailed notes were analyzed using thematic analysis and the constant comparative method (Braun and Clarke, 2006; Saldana, 2012). Two researchers first reviewed all data independently and identified broad themes that emerged. They then shared their initial findings, discussed their codes, and reviewed the data a second time. At the end of this second coding, the researchers worked to eliminate disagreements through conversation, and arrived at a set of findings guided by the research questions.

## Results

### RQ 1: Simulated Versus Enhanced Virtual Reality

Analysis (see Table 4) found that participants preferred enhanced experiences over the simulated experience, and all preferred the enhanced symphony over the campus tour. In part one, many participants (*n* = 7) perceived the simulated symphony as poor video quality (although all videos were 4 k resolution) and were disappointed by the lack of environmental immersion. Some (*n* = 3) thought the experience was no better (and, in some cases, worse) than watching a concert on television. Ken (FG1) explained, “You just feel like you’re watching a picture or video. I never saw anyone around me.” Gene (FG2) was also disappointed: “It felt like we were in the cheap seats.” Evelyn (FG2) agreed: “The picture was blurry. If you looked down, you could see the heads of the people in the audience, but just a little. It made it a little more realistic, but it was a fuzzy picture […] The sound sounded like a recording […] You miss a lot because you’re not there.” June (FG2) kept asking if the volume could be adjusted; she explained, “I don’t feel I heard the music very well […]”

**TABLE 4:** Summary of research findings.

**Research question**	**Associated findings**
RQ1: How do older adults, including PWD and CPs, perceive simulated and enhanced VR experiences?	Participants perceived *enhanced experiences* (i.e., experiences that exceeded the opportunities in real life) as more immersive and enjoyable when compared with *simulated*. They reported fewer complaints about the HMDs and visual/auditory issues when engaged with enhanced experiences
RQ2: To what extent do participants perceive salutogenic design qualities (e.g., sense of “being away”, social connection, and/or enjoyment) in the simulated versus enhanced VR experiences?	With enhanced experiences, participants reported escaping or feeling immersed in a new environment (e.g., Hal talked about “going away” to the symphony when experiencing the enhanced symphony VR environment). Participants also felt more connected to musicians and the conductor in the enhanced symphony, and they reported more enjoyment in the enhanced experiences
RQ3: Do perceptions and experiences of PWD differ from other participants?	PWD reported lower levels of immersion and engagement with the VR experiences. They also experienced greater technology challenges when compared with other participants
RQ4: What desires and expectations do older adults have for future VR experiences?	Participants desired enhanced experiences. They specifically asked for opportunities to engage in activities no longer available to them (like travel or outdoor recreation). They also saw VR as a potential avenue for connecting with friends and family across distance

In part two, participants were much more pleased with the enhanced symphony, where they were positioned on center stage and music direction varied according to instrument positionality. Additionally, participants had fewer complaints about audio-visual quality or HMD comfort. All participants in the last three focus groups had visual impairments (*n* = 6), yet only two reported issues seeing musicians and instruments. However, both participants noted that when images became unclear, they could adjust the headset to make them clear, demonstrating blurry images were not due to video quality and they were motivated to improve the visual experience.

All participants experienced the virtual campus tour. Participants in focus groups 1–3 who experienced the replication symphony preferred the campus tour (except one PWD who declined to participate in the tour); those in focus groups 4–6 all preferred the enhanced symphony over the tour. Leann (FG3) described her preference for the tour over the simulated symphony: “[It was] fascinating! It felt like you were right there, and you could see everything. When the handle came out right in front of you, I wanted to grab it!” Conversely, all participants (*n* = 6) who experienced the enhanced symphony preferred it over the tour. Betty (FG4) talked about her desire to spend more time at the symphony, “I preferred the symphony, because it was just that. And then you could do your looking [around], and you could ‘Oh, I want to see who’s directing?’ And you could turn around. Or you could ‘Oh, I want to see if [musician] is there!’ […] You could concentrate more on it.” Leann, the only participant to experience all three virtual experiences, changed her preference from the tour (FG3) to the enhanced symphony (FG4): “It’s so wonderful! It was very interesting and very exciting! It felt so real! You felt like you were part of the orchestra! I’d like to stay there for a longer time.”

### RQ 2: Perceptions of Salutogenic Design Qualities

Participants expressed positive distraction and greater engagement in both enhanced VR experiences and improved social connectedness in the enhanced symphony experience. Participants were less likely to comment about HMD discomfort or audio-video dissatisfaction during enhanced VR experiences, suggesting increased immersion. In the enhanced symphony, participants described a strong sense of social connectedness with musicians and the conductor. For example, Leann (FG4) talked about proximity and connection with the musicians, “Oh my goodness! [The musicians] are so close to me! Oh, this is wonderful!” Darla (FG6) watched the conductor —a perspective she could not experience at a live concert: “And to turn around and watch the director! I thought it was interesting because each director has their own hand signals.” Similarly, Hal (FG5) expressed his engagement, “The focus, the sound, the vision of being able to watch and observe the orchestra. You’re right there. They’re right in front of you. I wouldn’t change anything. It’s amazing!” As his focus group continued, Hal kept commenting on how “wonderful” the symphony was. Several times he joked about stealing a headset so that he could spend his free time “at the symphony,” demonstrating his ability to escape and feel fully immersed.

### RQ 3: Similarities and Differences in Experiences of People With Dementia

People with dementia participated in the first (Fred and Robert) and third (June) focus groups, which compared the simulated symphony and enhanced campus tour. At times, PWD echoed the experiences of other participants. For example, Fred talked about his desire for clearer images and more interactive environments after the replication symphony: “It’s not quite as interactive as I’d like.” At other times, specific challenges arose for PWD. For example, when asked about what she liked about the replication symphony, Jane explained that she could not remember much—a common occurrence resulting from dementia. In FG1, both Fred and Robert struggled to work the headset more than other participants. At one point, Fred expressed frustration, “I don’t think I’m doing anything right!” Eventually, Fred was able to experience the virtual campus tour, and described it as disappointing, “I’m really not comfortable with [the headset]. I’m not sure what I can get out of it. I heard some of the music loud enough, so it was easy to spot it and all that, but I didn’t know what to do with it. So, I got it moving around a little bit, but I didn’t feel like it was doing anything I asked it to do.” Similarly, Robert experienced the replication symphony after overcoming some initial hesitancy. He provided limited feedback and then declined to try the headset again. Robert was the only participant who declined a second virtual experience. In general, PWD expressed less sense of VR immersion and engagement and greater technology challenges.

### RQ 4: Desires and Expectations for Future Virtual Reality Experiences

After engaging in the VR experiences, participants provided suggestions for future experiences. In general, participants desired experiences aligning with salutogenic design principles that could increase their engagement beyond real-world limitations and allow greater personal control. For example, several (*n* = 4) participants who viewed the enhanced symphony noted that they could still see the symphony live; however, they could not view a performance from center stage. They all acknowledged the potential for VR to connect them with activities that they could not do. Participants talked about going surfing, skiing, or hiking: “There’s things I’ve never experienced that I would love to do still, you know, even at my age. [Skiing] would be exciting […]” (Sheila, FG2). Several (*n* = 7) participants also talked about using VR to drive or travel, activities they missed. A few (*n* = 4) talked about the potential to watch sporting events, as Leann (FG3) explained: “I think viewing sports would be great for people who are stuck in their homes and can’t get out.” In general, participants saw VR’s potential, and most were excited by opportunities to overcome their limitations in virtual spaces. Carl (FG5) talked about his desire to use VR to connect with his family: “[…] I have a lot of grandkids and children that live elsewhere, spread around. It’d be nice to actually see them.” Indeed, several participants talked about VR as a way to connect with others that would be more immersive than phone calls or text messages. Finally, one participant suggested greater control (i.e., improving self-efficacy) in the VR experiences: “I think what I would like is for each person to have more control [.] over what I was looking at. To stay longer on one thing and then maybe switch to another thing.” (Leann, FG4).

## Discussion

Research suggests that non-pharmacological interventions have salutogenic potential to delay cognitive decline and enhance quality of life among older adults (Davalos et al., 2019; Faw et al., 2021). There is a critical need to develop effective, accessible, and enjoyable interventions to help preserve cognition. Results from six focus groups exploring three VR experiences indicates important opportunities and challenges for developing VR as a salutogenic strategy for enhancing wellbeing among older adults. An important conclusion from this research is that VR is a viable intervention for older adults with and without dementia, and co-design processes may be critical for improving salutogenic design features. Importantly, co-design processes revealed that older adults perceived VR experiences positively but do not want virtual experiences that merely simulate real-world ones. Participants during the first three focus groups universally identified the replication symphony experience as underwhelming. Conversely, participants in the last three focus groups praised the enhanced symphony experience, describing it as immersive and exhilarating. It is also important to note that participants found the immersive symphony experience *more* engaging and enjoyable than the activities in the campus tour. Our long-term goal has been to recreate real-world symphony experiences, including walking into the concert hall, virtual interactions with audience members before the concert, etc. However, given participants’ desire for enhanced experiences, we believe continued co-design to explore their reactions to these simulated elements is essential in building out future VR experiences. Notably, PWD experienced less immersion and more technology challenges than others, suggesting co-design with PWD is both feasible and important for understanding barriers and opportunities specific to this population. Furthermore, co-design with PWD at different disease stages is needed to better understand how to design VR to enhance its salutogenic potential. Involving older adults as collaborators to co-design technologies at all stages (e.g., from pre-design through prototype testing and iteration, to product evaluation and impacts on wellbeing) is essential for improving salutogenic potential (Sumner et al., 2021). With this in mind, we make the following recommendations:

### Practical Suggestions for Future Virtual Reality-Enhanced Experiences With Older Adults

#### Engage Older Adults in the Co-design of Virtual Reality Experiences

Virtual reality interventions are viable for older adults; participants were willing and excited to use VR with assistance. As Hal (FG5) said, “I gotta tell ya, this is a winner for us seniors.” However, there remains little research about how to design engaging VR experiences for older adults. Prior to conducting the focus groups, we anticipated that participants would feel socially connected by “virtually” sitting in the audience during a symphony performance. However, this was not the case; social connection was fostered when video was captured from center stage. This highlights the value of early and frequent engagement with older adults and suggests co-design during pre-design phases may be useful for identifying VR experiences older adults would find most engaging.

#### Consider the Unique Needs of People With Dementia, Including How These Change With Disease Progression

Based on prior research, we anticipated that PWDs would have greater challenges with technology; however, we did not anticipate that their sense of immersion would be lower than others. Frustration with technology may have mediated engagement in VR. However, sense of immersion and engagement might also have been affected by stage of disease progression, suggesting more co-design research with PWD is needed to understand what types of salutogenic interventions may be beneficial. PWD may be particularly sensitive about being embarrassed if they find the VR experience confusing, meriting special design attention (Hodge et al., 2018; Lee et al., 2019).

#### Salutogenic Design Principles May Be Useful for Creating Virtual Reality Experiences

Immersive VR involves complicated aspects of film and environmental design. Salutogenic design is a human-centered, evidence-based approach to designing environments to promote wellbeing. Our study considered only a few aspects of salutogenic design (positive distraction, environmental engagement, and social connectedness). Future projects may find salutogenic design a useful framework and, in turn, findings from VR research (where experimental control is more feasible) may help to inform salutogenic design strategies for real-world settings.

#### Consider Technology Access, Affordances, and Limitations

There were several practical challenges with our study’s headsets. At least three participants noted that the headsets felt heavy, forcing them to limit their time in the headset. Additionally, all participants hesitated to use the headsets without guidance, indicating a barrier to adoption. Nevertheless, several participants mentioned interest in being able to check out VR headsets from the library and in buying a headset for personal use. From a research standpoint, VR is a beneficial tool as it puts participants in controlled environments and gives researchers the ability to generate varied and immersive stimuli for them. VR technology is rapidly evolving, and, since our pilot study, new, lighter headsets have come to market with improved graphics. Additionally, a viable VR experience for older adults would require significant time to train participants to use HMDs without assistance. Studies that have included training protocols found improved VR comfort and usability (Optale et al., 2010; Wen et al., 2018), thus investing time and resources into training older adults may be beneficial in overcoming VR adoption limitations.

### Limitations and Directions for Future Research

While our study presents valuable data, it is not without limitations. First, these results represent a small, homogenous sample drawn from one region of the United States. To fully capture the potential of VR interventions, researchers should test VR designs across a broader sample, including individuals from different racial and cultural backgrounds. Second, this study used three specific VR experiences. The fact that our study used three designs (and made adjustments based on participant feedback during the project) is both a strength and a limitation. It allowed our participants more than one experience to reflect on when sharing their impressions. However, these experiences are not representative of the diverse VR content available. As such, researchers should explore how older adults respond to different VR experiences, especially by engaging them early in co-design. We hope to see future projects co-create VR experiences that are tailored to participants’ unique background and personal preferences, as this might be particularly valuable for PWD. Additionally, PWD and CPs only experienced the simulated symphony and campus tour, giving us limited insight regarding how they might respond to other enhanced experiences (like the enhanced symphony). Another limitation was the fact that PWD participants were not tested for their dementia stage. As dementia is a degenerative condition, it is possible that PWD at earlier stages might experience VR differently and appreciate varying VR dimensions than PWD at more advanced stages. Continued co-design with PWD can attend to these issues more carefully, working to develop recommendations designed to highlight best VR practices across the dementia spectrum.

## Conclusion

The present study used salutogenic design principles and co-design methods to explore older adults’ reactions to three VR experiences. Across focus groups, participants preferred enhanced experiences and benefited from the salutogenic design properties of social connection, immersion, and engagement. Participants expressed their desires for enhanced experiences in future VR interventions. PWD and CPs experienced greater technology challenges and lower levels of immersion, indicating that additional co-design research with these populations is needed to produce to more effective interventions that attend to their unique needs.

## Data Availability Statement

The datasets generated for this study are available on request to the corresponding author.

## Ethics Statement

The studies involving human participants were reviewed and approved by Colorado State University Institutional Review Board. The patients/participants provided their written informed consent to participate in this study.

## Author Contributions

MF and TB conducted all the focus groups and analyzed the focus group data. MF and LM secured funding for the project. All authors contributed to the conception and design of the study, wrote, read, and approved the final manuscript version.

## Conflict of Interest

The authors declare that the research was conducted in the absence of any commercial or financial relationships that could be construed as a potential conflict of interest.

## Publisher’s Note

All claims expressed in this article are solely those of the authors and do not necessarily represent those of their affiliated organizations, or those of the publisher, the editors and the reviewers. Any product that may be evaluated in this article, or claim that may be made by its manufacturer, is not guaranteed or endorsed by the publisher.

## Footnotes

1 Cognitive abilities vary across adulthood. For instance, executive functions and working memory usually peak in the 3rd decade of life and decline thereafter. This decline becomes especially prominent from the 7th decade of life onward.
